# Immune Regulation in Atrial Cardiomyopathy

**DOI:** 10.31083/RCM26897

**Published:** 2025-04-30

**Authors:** Si-ming Tao, Man Yang

**Affiliations:** ^1^Department of Cardiology, The Affiliated Hospital of Yunnan University, 650021 Kunming, Yunnan, China; ^2^Department of Cardiology, The First People’s Hospital of Dali, 671000 Dali, Yunnan, China

**Keywords:** atrial cardiomyopathy, remolding, fibrosis, immune regulation

## Abstract

Clinical observations have shown that cases of stroke or thromboembolism are not uncommon even in the absence of atrial fibrillation, suggesting that atrial fibrillation is a delayed marker of atrial thrombus formation. Atrial cardiomyopathy (ACM) is a pathophysiological concept characterized by atrial substrate and functional abnormalities closely associated with atrial myopathy, atrial enlargement, and impaired ventricular diastolic function. It is an independent factor for thromboembolic stroke, increasing the risk of serious complications such as atrial fibrillation, heart failure, and sudden cardiac death. ACM is likely to be a potential cause of embolic stroke, especially cryptogenic stroke, and early identification of patients at high thromboembolic risk is essential to guide anticoagulation therapy. Although the pathogenesis of ACM has not been fully elucidated, prospective mechanism-based studies have revealed the important role of activated cardiac immune cells along with inflammatory responses, oxidative stress, and other factors in its progression. Exploring the role of immune regulation in the pathogenesis of ACM provides new insights into the underlying mechanisms of cerebrovascular events of cardiac thromboembolic origin. This review summarizes the mechanisms by which immune regulation is involved in the progression of ACM and provides useful insights for future clinical diagnosis and treatment.

## 1. Introduction

Atrial fibrillation (AF) is a globally prevalent, age-related arrhythmia and the most serious complication associated with it, significantly contributing to the risk of thromboembolism [1]. However, 30% of ischemic strokes are covert, and the reasons for this are unclear. Additionally, there is a lack of time association between the onset of AF, which poses significant challenges in clinical diagnosis and treatment [2, 3].

Recent studies have highlighted the close interaction between atrial cardiomyopathy (ACM), AF, and stroke, suggesting that ACM may serve as a critical substrate for the development of AF and subsequent thromboembolic events [4]. Several factors contribute to the pathophysiology of ACM, including aging [5], inflammation [6], oxidative stress [7], epicardial fat infiltration [8], and alterations in blood flow shear stress [9]. These factors promote processes such as fibrosis, atrial remodeling, and thrombus formation. The intricate interplay of these mechanisms not only leads to the progression of ACM but also increases the risk of developing AF and stroke, creating a detrimental feedback loop [10].

Emerging research indicates that immune cells and immune-active molecules play pivotal roles in the pathophysiological mechanisms of atrial remodeling and myocardial fibrosis, participating in many important inflammatory reactions, maintaining internal environment, homeostasis, and influencing metabolic processes [11, 12]. Understanding the immunomodulation-mediated pathophysiological mechanisms in ACM may provide valuable insights into the progression of AF substrates and the mechanisms underlying atrial thrombosis.

## 2. Atrial Cardiomyopathy

ACM is defined as a collective term for a group of diseases that affect the structure, configuration, contraction, or electrophysiological function of the atria and may lead to related clinical manifestations [4]. To date, there are no standardized diagnostic criteria for ACM, with crucial diagnostic information primarily derived from clinical history and responses to rhythm and rate control therapies. Atrial tissue biopsy serves as the gold standard for pathological diagnosis and classification of various types of ACM. However, invasive examinations are limited by diagnostic follow-up and lack clinical operability. Cardiac magnetic resonance imaging, as a repeatable diagnostic tool, is utilized to identify and quantify the extent of atrial fibrosis, becoming the gold standard for describing cardiac chamber structure and function [13]. In clinical practice, analyzing electrocardiogram P-wave morphology (P-wave index) reflects the potential correlation between atrial remodeling and ischemic stroke [14]. Transthoracic echocardiography (two-dimensional (2D) imaging, pulse wave Doppler, two-dimensional speckle tracking echocardiography, strain and strain rate imaging) combined with cardiac computed tomography accurately and non-invasively diagnose ACM and predict arrhythmia recurrence after pulmonary vein isolation [15]. Four-dimensional (4D) flow cardiac magnetic resonance imaging is a novel non-invasive method for evaluating atrial hemodynamics, providing comprehensive detection and quantification of blood stasis in the atria and atrial appendages [16]. Markers of ACM, such as the P-wave terminal force in electrocardiogram lead V1 (PTFV1), left atrial dimension (LAD), N-terminal pro-B-type natriuretic peptide (NT-proBNP), and excessive Atrial Ectopy can predict the occurrence of ischemic stroke in the general population [17, 18, 19, 20, 21]. New technologies such as genetic testing, immunomics, and single-cell analysis can provide a basis for the diagnosis of ACM, but further research is needed to obtain more precise diagnosis and treatment.

## 3. Atrial Cardiomyopathy and its Relationship with Atrial Fibrillation and Thromboembolic Stroke

**Fig. 1.**
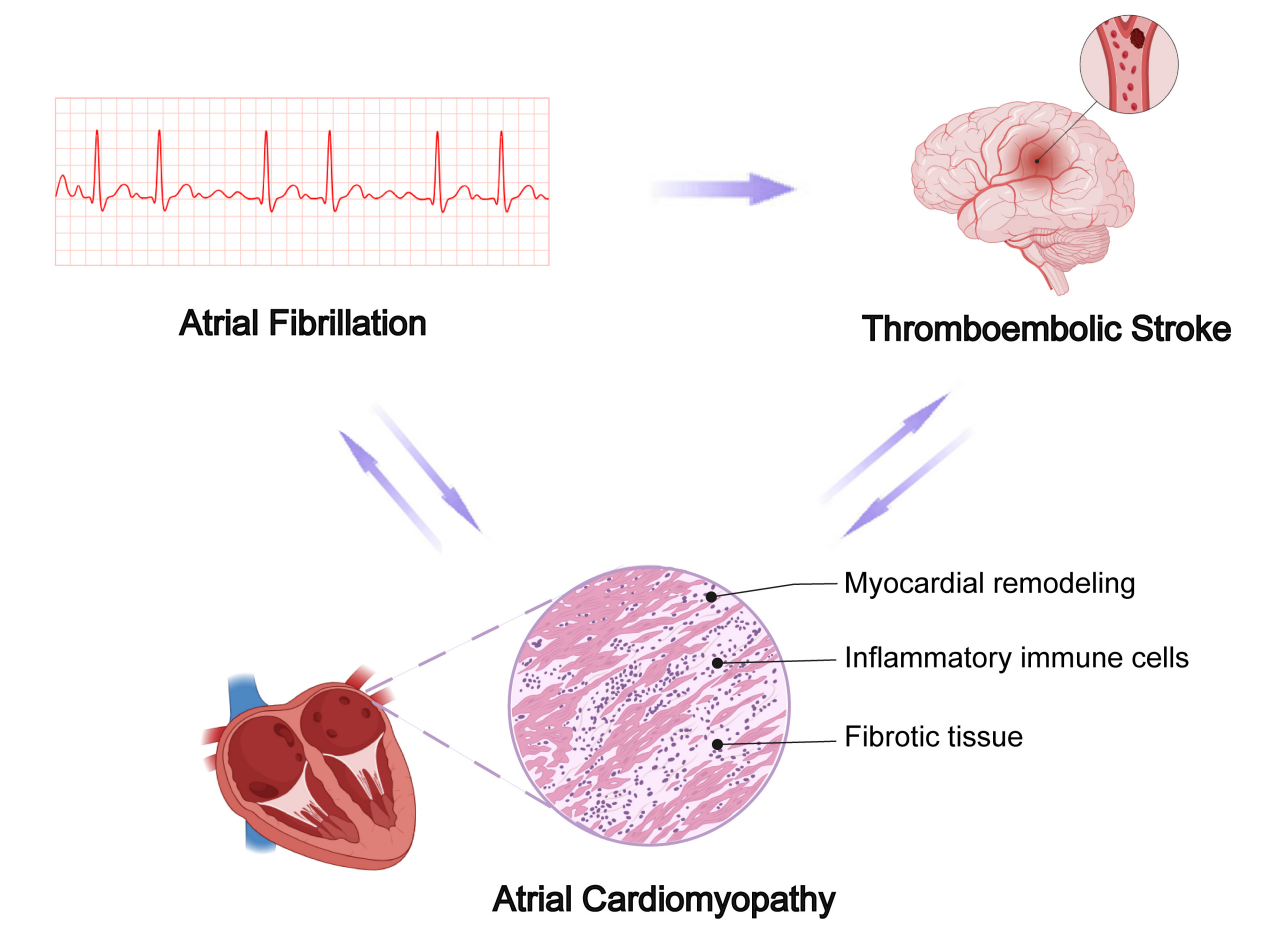
**Pathophysiology of atrial cardiomyopathy in relation to atrial fibrillation and thrombosis**. The figures in the manuscript were edited using Adobe Illustrator 2020 (Adobe Inc. San Jose, CA, USA). Additionally, some image materials were sourced from the open-access website Biorender (https://www.biorender.com).

ACM encompasses various pathophysiological manifestations, including atrial remodeling, fibrosis, mechanical and electrical dysfunction, and a prothrombotic state. Multiple risk factors contribute to the development of ACM, such as atrial arrhythmias, hypertension, heart failure, diabetes, obesity, inflammation, obstructive sleep apnea, endocrine disorders, autoimmune diseases, genetic factors, aging, and psychiatric disorders [22]. Among these, AF is the primary clinical manifestation associated with ACM, creating a vicious cycle where ACM both influences and results from AF [23]. ACM, particularly when accompanied by AF, is linked to an increased incidence of thromboembolic events [24]. However, the temporal dissociation between AF and thromboembolic events suggests that ACM may represent an independent and progressive disease process [25]. Animal studies infer that the association between AF and stroke is due to bidirectional causality between ACM and a prothrombotic state, with mutual reinforcement and common underlying causes [24]. Inflammatory processes associated with AF can lead to endothelial damage and immune factor activation, which may directly contribute to thrombus formation [26]. The degree of fibrosis in the left atrium is critical in promoting AF and thrombotic events [27]. Additionally, inflammatory stimuli can impair endothelial function in atria, further heightening the risk of thrombosis [24]. The activation of immune cells, particularly T cells, monocytes, and macrophages, under pathological conditions triggers an inflammatory response and facilitates fibroblast recruitment, leading to myocardial fibrosis [28]. Investigating the immune-inflammatory responses related to ACM may clarify the connections among ACM, AF, and embolic stroke, potentially guiding anticoagulation strategies and immunotherapy (Fig. 1).

## 4. Atrial Cardiomyopathy: Pathophysiology

### 4.1 Atrial Remodeling

Atrial remodeling is a response to persistent structural and functional changes in atrial tissue, driven by factors such as pressure overload, inflammation, oxidative stress, and electrical stressors. This remodeling serves as the substrate for true ACM [23]. Atrial remodeling is based on three main pathophysiological processes: structural remodeling, electrical remodeling, and functional remodeling [23]. Atrial structural remodeling is the result of increased interstitial fibrosis accompanied by changes in cardiac structure and atrial dilation, which is the main pathological manifestation of atrial functional deterioration [29]. Regardless of left atrial size, once atrial remodeling occurs, it leads to loss of atrial mechanical function [30]. Abnormal atrial contractile function in ACM leads to heterogeneous atrial conduction and triggers AF [20]. During AF, abnormal atrial mechanical contractile function initiates and sustains a self-perpetuating vicious cycle. Early myocardial remodeling (exposure <1 week) is a compensatory mechanism that is reversible and aligns with the Frank-Starling curve, enhancing atrial myocardial contractility. Over time, cellular, electrical, and autonomic nerve function impairment becomes permanent and decompensated [14]. Inflammatory events alter the cardiac microenvironment, promoting fibrosis progression by various cardiac cells (such as fibroblasts, endothelial cells, inflammatory and immune cells), soluble factors, and extracellular matrix (ECM), leading to atrial tissue remodeling. While cardiac fibroblasts are considered crucial regulators of ECM remodeling, inflammation plays a vital role in promoting cardiac fibrosis [31]. Therefore, deciphering the role of immune cells in the cardiac microenvironment may offer novel targeted strategies for fibrotic remodeling [32].

### 4.2 Fibrosis

Fibrosis is a prominent histopathological feature and mechanical abnormality resulting from sustained early AF; however, successful ablation to eliminate AF does not prevent fibrosis progression. Evidently, atrial matrix fibrosis may be both a consequence and a cause of arrhythmias [33]. Fibrosis is a hallmark pathological finding visible in structural remodeling and may serve as the pathological basis for the occurrence and persistence of AF in patients with ACM. A study emphasizes the correlation between ACM and other coexisting pathologies, with fibrosis being the most interconnected pathological mechanism [13]. Mays H *et al*.’s study [34] indicates that even in patients with sinus rhythm, increasing atrial fibrosis is associated with a higher incidence of stroke. Despite increasing research on fibrosis, its mechanisms remain inadequately explained, hindering progress in targeted anti-fibrotic drug research [35]. New study indicates that complex interactions between immune cells, fibroblasts, and other non-immune/host-derived cells are considered the primary drivers of cardiac fibrosis and inflammation has emerged as a new target for anti-fibrotic therapy [36].

## 5. Immune Regulation is Involved in the Mechanism of Atrial Cardiomyopathy

### 5.1 Immune System Abnormalities

**Fig. 2.**
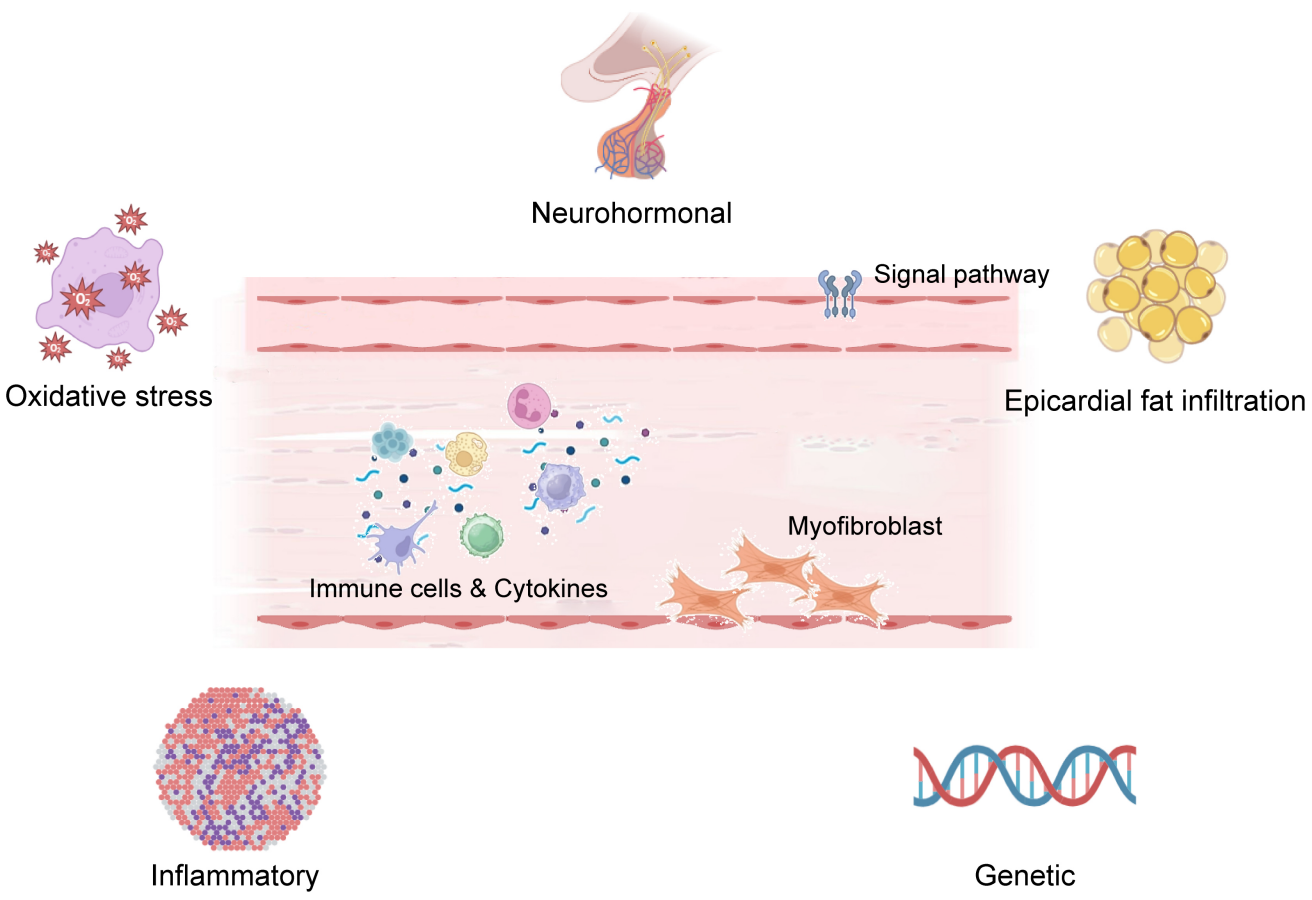
**Immune regulation is involved in myocardial remodeling and fibrosis**. The figures in the manuscript were edited using Adobe Illustrator 2020. Additionally, some image materials were sourced from the open-access website Biorender (https://www.biorender.com).

Immune system abnormalities may represent early events in the development of fibrosis, particularly in the context of atrial remodeling processes [12, 37]. These abnormalities involve mechanisms such as ion channels, neurohormones, inflammatory responses, gene expression, and alterations in the extracellular matrix [38]. Identified immune cells in the heart, such as T lymphocytes, macrophages, dendritic cells, granulocytes, and mast cells, participate in the fibrotic process [10]. These activated immune cells highly express factors that regulate inflammation and fibrosis, promoting fibroblast activation (Fig. 2). Fibroblasts synthesize a large amount of collagen fibers, accelerating tissue repair in damaged tissues, leading to cardiac fibrosis and increasing the risk of heart failure and atrial dysfunction [10]. Specific immune cells play crucial roles in the fibrotic process associated with ACM. T lymphocytes, particularly CD4+ T cells and CD8+ T cells, are known to contribute to the inflammatory milieu within the atria. They can produce pro-inflammatory cytokines that not only amplify the inflammatory response but also promote fibroblast activation and proliferation. This activation leads to increased collagen synthesis, exacerbating fibrosis and potentially facilitating the onset of AF [39]. Macrophages, being the predominant leukocytes in the heart and a significant supplier of cytokines, have a well-documented role in repair and fibrosis during the initial phases of cardiac injury [40]. Macrophages exhibit remarkable plasticity, enabling them to adopt various phenotypes based on the local microenvironment [41]. The diversity of macrophage populations—spanning from pro-inflammatory (M1) to anti-inflammatory (M2) phenotypes—can either alleviate or worsen the fibrotic process, ultimately affecting the progression of atrial dysfunction [41]. IgE belongs to a class of immunoglobulins, and the IgE-FcepsilonR1 pathway has been implicated in pathological cardiac remodeling, highlighting the complex interplay between immune responses and structural changes in the heart [42]. Immunotherapies such as monoclonal antibodies, cell therapy, and small molecule inhibitors are attractive but challenging targets for regulating cardiac homeostasis and inhibiting cardiac remodeling.

### 5.2 Abnormal Activation of the Neurohormonal System

Under the interaction of various risk factors, the renin-angiotensin-aldosterone system (RAAS) and neurohormones such as atrial natriuretic peptide are activated, participating in the progression of ACM [35]. Aldosterone has been shown to induce fibrosis in the liver, kidneys, and heart diseases, with fibroblasts and myofibroblasts playing a central role through RAAS activation in the heart. More evidence suggests that angiotensin II (Ang II) plays a crucial role in promoting atrial remodeling, with Ang II-mediated atrial electrical and structural remodeling developing earlier and more extensively in the left atrium compared to the right atrium [43]. Activation of RAAS leads to the generation of reactive oxygen species (ROS) by Ang II, causing calcium handling abnormalities that may induce myocardial cell hypertrophy, endothelial dysfunction, and myocardial fibrosis [22]. Ang II binds to angiotensin receptor 1 (AT1-R), regulating fibrosis formation and inflammatory responses through various signaling pathways such as transforming growth factor-beta (TGF-β), Smad2/3, C-X-C motif chemokine receptor 2 (CXCR2), and nuclear factor kappa-B (NF-κB) [36, 43]. Atrial natriuretic peptide (ANP) and B-type natriuretic peptide (BNP) secreted by the atria are regulated by a complex network of endocrine, neural, and immune systems and are important regulators of homeostasis [44]. Elevated ANP levels may reflect increased atrial filling pressure and dysfunction [23]. Elevated NT-proBNP levels are associated with thromboembolic events related to the atria or other pathways unrelated to AF, aiding in predicting ischemic cerebrovascular events in the general population [45]. The study has show that myocardial calcitonin levels are six times higher in AF patients than in normal subjects and are involved in pathways related to fibrogenesis, infection and immune response, and transcriptional regulation, and that atrial cardiomyocytes are also an active source of calcitonin; restoration of impaired myocardial calcitonin-calcitonin receptor signaling may offer a new strategy to inhibit atrial remodeling [46]. Different neurohormonal-immune interactions can show favorable or maladaptive responses in cardiac remodeling.

### 5.3 Oxidative Stress

Oxidative stress is considered a core component in the process of atrial remodeling [47]. Factors such as diabetes, heart failure, and aging (Congestive heart failure/left ventricular ejection fraction ≤ 40%, Hypertension, Age ≥ 75 [2 points], Diabetes mellitus, prior Stroke/transient ischemic attack/thromboembolism [2 points], Vascular disease, Age 65–74, Sex category female, CHA_2_DS_2_-VASc score) can alter the oxidative stress pathways within endothelial cells, leading to atrial reconstruction and promoting arrhythmias by increasing the formation of reactive oxygen species (ROS) and reactive nitrogen species (RNS) [48]. Oxidative-reductive mechanisms regulate various immune functions, including but not limited to macrophage and Th cell polarization, phagocytosis, and the production of pro-inflammatory and anti-inflammatory cytokines [49]. Oxidative stress affects the performance and survival of individual immune cells, exacerbating inflammation and fibrosis. Therefore, the role of ROS/RNS in ACM is particularly emphasized. Mitochondria are one of the main sources of ROS and exhibit significant structural and morphological changes during AF, such as swelling and loss of cristae structures. When not effectively compensated by antioxidants, endogenous ROS may have potential harmful effects on cell structure, including nuclear DNA and mitochondrial molecules [47]. Metabolic remodeling pathways induced by DNA damage are fundamental to atrial and ventricular cardiomyopathy [48], providing an opportunity for new therapeutic strategies based on elucidating the role of DNA damage mechanisms.

### 5.4 Inflammatory Response

Inflammatory responses induce cell damage, apoptosis, mediate structural remodeling and fibrosis, playing a crucial role in cardiac fibrosis [29]. Damaged or apoptotic myocardial cells produce numerous inflammatory factors such as C-reactive protein (CRP), tumor necrosis factor-alpha (TNF-α), interleukin-2, interleukin-6, interleukin-8 (IL-2, IL-6, IL-8), and monocyte chemoattractant protein-1 (MCP-1), promoting fibroblast proliferation [50]. CXCR2 regulates monocyte/macrophage entry into cardiac tissue and further development of AF. Conversely, inhibiting the CXCR2-MAPK (mitogen-activated protein kinase), NF-κB, TGF-β/Smad2/3 pathways significantly weakens monocyte/macrophage infiltration into the atrium, inducing AF and atrial remodeling [51]. C-X-C motif chemokine ligand 12 (CXCL12) recruits smooth muscle progenitor cells and endothelial progenitor cells to promote tissue repair or recruit inflammatory cells to some extent to exert pro-inflammatory effects [52]. Evidence suggests that non-coding microRNAs (miRNAs), long non-coding RNAs (lncRNAs) serve as epigenetic biomarkers for evaluating inflammatory and fibrotic signal transduction [31]. LncRNAs prevent cardiac remodeling and fibrosis by regulating macrophage (M1/M2) inflammatory functions [40]. Bhlhe40, a member of the basic helix-loop-helix family E40, is an essential transcription factor implicated in pathological cardiac remodeling by regulating inflammation signaling pathways associated with atrial remodeling and progression of AF [53]. These biomarkers and signaling molecules in the progression of ACM may be potential targets for intervention, aiding in predicting the risk of AF in patients. The complexity of mechanisms necessitates future research exploring the effects of simultaneously intervening in multiple pathways to comprehensively target atrial remodeling.

### 5.5 Epicardial Fat Infiltration

Infiltration of epicardial fat has been recognized as an essential factor in understanding how AF substrates develop. In clinical settings associated with atrial dilation, epicardial activation is a chronic process contributing to the progression of atrial remodeling [4]. The epicardium is reactivated during the formation of ACM, giving rise to a subset of cells that perform specific functions aiding in fat infiltration beneath diseased atrial epicardium [8]. Factors secreted by adipocytes can transform into pro-inflammatory and pro-fibrotic signals stimulating myofibroblast differentiation, leading to significant fibrosis in epicardial fat tissue and myocardium [54]. Suffee *et al*. [8] used single-cell RNA sequencing to analyze atrial extracellular cells harvested from a rat model of ACM, indicating that extracellular expansion is an early event in the formation of ACM. The study revealed the specificity of extracellular-derived cells from adipocytes to fibroblasts and the biological basis of chronic atrial myocardial remodeling. Interestingly, the related research also found that immune cells in the myocardium may be involved in the transition from adipose infiltration to fibrotic infiltration [55].

### 5.6 Genetic Biomarker

Genetic variants that are functionally active in the atria and contribute to atrial development and/or maintenance of atrial electrical, structural, and metabolic properties may directly contribute to or alter susceptibility to ACM [56]. Primary fibrotic atrial cardiomyopathy (PF-ACM) is characterized by primary atrial fibrosis with an increased risk of arrhythmogenicity and stroke without atrial muscle involvement [57]. However, the pathogenesis of PF-ACM remains unclear and needs to be explored urgently. Zhu Y’s team [57] analyzed the genotype-phenotype correlation of 33 patients with PF-ACM by high-throughput sequencing, and found that 21 (63.6%) patients carried 33 cardiovascular disease-associated gene variants, which suggests the contribution of genetics to ACM. Mutations in the *NPPA* gene were also mentioned in the consensus on ACM, which is classified as an autosomal recessive form of ACM [58]. However, the genetic investigation of ACM is still in its infancy. With the development of molecular immunology and the application of emerging technologies, such as through the use of integrated bioinformatics methods and machine learning algorithms combined with multi-omics techniques, many gene expression profiles have been applied to study the distribution of immune cells and the critical role of genetic biomarkers in disease progression [59]. Elucidating the immune mechanisms of genetic genes in ACM may contribute to targeted prevention and individualized treatment of ACM.

## 6. Conclusions and Future Perspectives

The clinical value of ACM lies in identifying high-risk patients for thrombotic risk independent of AF diagnosis, guiding anticoagulation strategies, improving atrial arrhythmias and treatment of patients with unexplained thromboembolic events. The actual management of ACM is based on controlling risk factors, stroke prevention, arrhythmia treatment, heart rate control, and heart failure prevention. Compared to the traditional risk factors used for CHA_2_DS_2_-VASc, early identification of ACM theoretically can benefit from anticoagulant therapy [25, 60]. Catheter ablation for AF is the most effective rhythm control strategy to interrupt the vicious cycle between AF and ACM, preventing the progression of ACM [61, 62]. Cardiac resynchronization therapy (CRT) can partially reverse atrial remodeling in patients with heart failure with a reduced ejection fraction (HFrEF) [63]. Upstream therapies (statins, mineralocorticoid receptor antagonists, angiotensin-converting enzyme inhibitors/angiotensin II receptor blockers (ACEI/ARB), and omega-3 polyunsaturated fatty acids), anti-inflammatory therapy, antioxidant stress therapy, antifibrotic therapy, and immunotherapy are emerging options for the treatment of ACM [64, 65]. Furthermore, technological advancements including new models, single-cell techniques, and gene editing can provide new insights into the pathogenesis of fibrotic diseases and the development of therapeutic drugs [31]. Screening for ACM could be crucial in identifying at-risk patients. Early detection may allow for the initiation of risk reduction strategies, which could help prevent adverse outcomes associated with ACM. Translating the latest basic research and clinical findings into mechanism-based therapies to inhibit or even reverse atrial remodeling and fibrosis is the future direction of exploration in ACM.
